# Understanding the Feasibility to Implement Schistosomiasis Elimination Project Under China-Zimbabwe Cooperation: A Pilot Study Protocol

**DOI:** 10.1007/s44197-025-00418-6

**Published:** 2025-05-26

**Authors:** Yingjun Qian, Nicholas Midzi, Shizhu Li, Masceline Jenipher Mutsaka-Makuvaza, Shan Lv, Wei Ding, Zhiqiang Qin, Hongmei Li, Jie Zhou, Ling Tang, Changlian Li, Xinling Yu, Liang Shi, White Soko, Isaac Phiri, Cremance Tshuma, Munyaradzi Dobbie, Xiao-Nong Zhou

**Affiliations:** 1 National Institute of Parasitic Diseases at Chinese Center for Disease Control and Prevention (Chinese Center for Tropical Diseases Research); National Key Laboratory of Intelligent Tracking and Forecasting for Infectious Diseases; Key Laboratory on Parasite and Vector Biology, Ministry of Health; WHO Centre for Tropical Diseases; National Center for International Research on Tropical Diseases, Ministry of Science and Technology, Shanghai, 200025 China; 2 National Institute of Health Research, Ministry of Health and Child Care, Harare, Zimbabwe; 3 University of Rwanda, Kigali, Rwanda; 4 Université Côte d’Azur, ESPACE UMR 7300, Nice, France; 5 Hunan Institute of Schistosomiasis Control, Yueyang, China; 6 Jiangsu Institute of Parasitic Diseases, Wuxi, China; 7 Epidemiology and Disease Control, Ministry of Health and Child Care, Harare, Zimbabwe; 8 Mashonaland Central Province, Ministry of Health and Child Care, Bindura, Zimbabwe; 9 Public Health Division, Ministry of Health and Child Care, Harare, Zimbabwe

**Keywords:** China, Zimbabwe, Schistosomiasis, Elimination, Africa, Tropical disease

## Abstract

**Background:**

Schistosomiasis is one of the major neglected tropical diseases in Africa, accounting for approximately 90% of the global burden. In Zimbabwe, *Schistosoma haematobium* and *S. mansoni* infections are endemic. Although mass drug administration has been carried out among school-aged children, other interventions at the national level remain incomplete. China has established a public health cooperation mechanism with African countries targeting schistosomiasis and other infectious diseases. This study aims to conduct a pilot study to provide a methodological reference for large-scale surveys in similar settings.

**Method:**

This pilot study served as an entry point for China-Africa cooperation in schistosomiasis control. A combination of cross-sectional studies and snail surveys was used. The survey was carried out in 14 villages of Chevakadzi ward in Zimbabwe. Households were selected through simple random sampling for the study. Fecal and urine samples were tested in the laboratory to diagnose schistosomiasis. Meanwhile, a capacity and needs assessment was conducted to understand the current situation of local disease control strategies.

**Discussion:**

This study is expected to obtain important epidemiological information and indicators regarding the transmission of schistosomiasis at the sub-district level, providing a basis for judging the feasibility and practicality of large-scale China-Zimbabwe cooperation investments. The research results will also offer references for policy-making and the update of prevention and control strategies, contributing to schistosomiasis control in Zimbabwe. However, the study has limitations such as limited funding and difficulties in cross-border drug registration.

**Supplementary Information:**

The online version contains supplementary material available at 10.1007/s44197-025-00418-6.

## Background

Ancient yet neglected, schistosomiasis (SCH) is a vector-borne and water-borne zoonotic parasitic disease amongst a diverse group of ‘Neglected Tropical Diseases’ (NTDs) [1–3], caused by the flatworms of genus *Schistosoma* and can lead to acute and chronic conditions. Humans contract SCH through contact with infested freshwater sources, where cercaria, the infectious free-swimming larvae of schistosome penetrate the skin. SCH is recognized as a disease of poverty because it is generally prevalent in low-income, remote communities with inadequate access to clean water, basic sanitation and health care. This situation exacerbates its impact on impoverished populations and keep them trapped in generational cycles of poverty. Of the 5 species affecting human health, *Schistosoma haematobium* causes a urogenital form of disease while *S. mansoni*, *S. japonicum*,* S. mekongi and S. intercalatum* are responsible for intestinal and hepatic SCH [4]. Alternatively known as bilharziasis, SCH mainly refers to urogenital and intestinal forms caused by the two major species of *S. haematobium* and *S. mansoni*,* which are* most prevalent in sub-Saharan Africa [2, 5].

Globally, human SCH has been endemic in 78 countries with at least 90% of global disease burden in Africa, where the highest morbidity and mortality occur [6, 7]. According to the Global Burden of Disease estimation, despite increasing visibility and continuous global investment, SCH accounted for 1.5 million years lived with disability worldwide in 2016 [8–10]. Moreover, COVID-19 has further increased the uncertainty to achieve targets set by 2030 NTD roadmap, particularly in Africa [11–13, 14]. The disease compromises public health by contributing to malnutrition in children, preventing them from attending school, impairing cognitive development, and increasing high risk of getting HIV especially among women [2, 7, 15, 16]. Despite the availability of effective interventions, SCH remains the second to malaria in terms of its public health significance [8, 17, 18]. Professional and domestic activities such as farming, bathing, washing, and playing expose local dwellers to infection [19–22]. The World Health Organization (WHO) advocates for preventive chemotherapy (PC) through mass drug administration (MDA) with praziquantel to all at-risk populations, including pre-school-aged children, school-aged children, adults in endemic areas, and entire communities in highly endemic areas. A single prevalence threshold determines the frequency of treatment, and cross-sectoral approaches are integrated into the strategy [23]. However, WHO estimated that in 2021 only 30% of people requiring treatment were reported to be treated, highlighting a huge gap between the actual needs and provision of medicines [24]. Apart from PC, a comprehensive intervention to control and eliminate SCH has been advocated, encompassing access to safe water, improved sanitation and hygiene, education and snail control [23, 25].

In 2000, the inaugural ministerial conference of Forum on China-Africa Cooperation (FOCAC) was held in Beijing, fostering China and Africa public health cooperation. The 2018 FOCAC Summit adopted the Beijing Action Plan (2019–2021), emphasizing eight initiatives to strengthen ties, including disease control programs in Africa focused on malaria, HIV/AIDS, and SCH [26, 27]. Within this framework, a China-Zimbabwe SCH cooperation was established [28].

Zimbabwe has a long history of SCH [29]. *S. haematobium* and *S. mansoni* are the two schistosome species of major public health importance leading to hospital admissions in the country [30–33]. Previous studies showed the disease pattern and its focal transmission especially in northeastern areas [31–33]. The most recently conducted national survey showed 22.7% prevalence with over 3 million population affected nationwide in all provinces [34]. Based on the results, Zimbabwe has implemented mass praziquantel treatment for SCH targeting school-aged children for six consecutive years from 2012 to 2017. However, MDA was suspended from 2018 to 2019 for the impact assessment survey. Moreover, the planned MDAs in 2020 did not occur due to the COVID-19 pandemic that lasted until 2022 [35, 36]. However, the provision of praziquantel relies extremely on donor support in African countries [37, 38]. Another control strategy of snail control by molluscicide was proved to be effective in Zimbabwe, but was not sustainable because of logistics, finance, and environment factors [31, 39]. To present, no other complementary strategies have been embraced into the national policy of SCH control and elimination. On the other hand, China was found endemic with SCH since the early 20th century and has implemented rigorous strategies to control and eliminate SCH [40–42]. The control of SCH in China is an ever-uninterrupted phased campaign moving from morbidity control, infection control, transmission control to transmission interruption, elimination and eradication based on national criteria [43–45]. The control efforts were driven by snail control in the very early stage, followed by MDA [40]. Since 2004, the strategy has centered on infection sources, and has been integrated into ‘One Health’ approach with significant success [46, 47–51]. Praziquantel-based chemotherapy, widely used in China with slight side effects, has been effective in controlling transmission [52]. Encouragingly, a study in Zanzibar showed that China-made praziquantel was equally efficacious as Merck KGaA’s in treating *S. haematobium* [53]. Moreover, China has never stopped the research and development to eliminate SCH, incorporating innovations in diagnostics, interventions, modelling, and strategic policies, adapting to the decreasing infections [54–59]. Many of these advancements are now routine and hold promise to other endemic countries [26, 60–62].

In this context, a pilot study was put forward as a starting point for the China-Africa cooperation on SCH control and elimination, aiming to study the feasibility of a large-scale project with Zimbabwe. Herein, we outline the protocol for the study.

## Methods

### Study Area

Zimbabwe is a landlocked country in the southeast of Africa. The major rivers of Zimbabwe include the Zambezi, Limpopo, Sabi, and Runde. The climate is generally sub-tropical, with a hot, humid, and rainy season from mid-November to March, and a dry season from April to mid-November. Zimbabwe boasts of 8 rural based provinces and 2 metropolitans. According to the 2010 national SCH and soil transmitted helminthiasis (STH) baseline survey, Shamva district in Mashonaland Province carried the highest prevalence of SCH in the country [34]. There are 29 wards (sub-districts) in Shamva district. Among these, Chevakadzi ward has never been mapped for SCH. This ward has abundant water contact sites, including a dam, that are favorable for snails. The population size there is appropriate for conducting a baseline study.

**Fig. 1:**
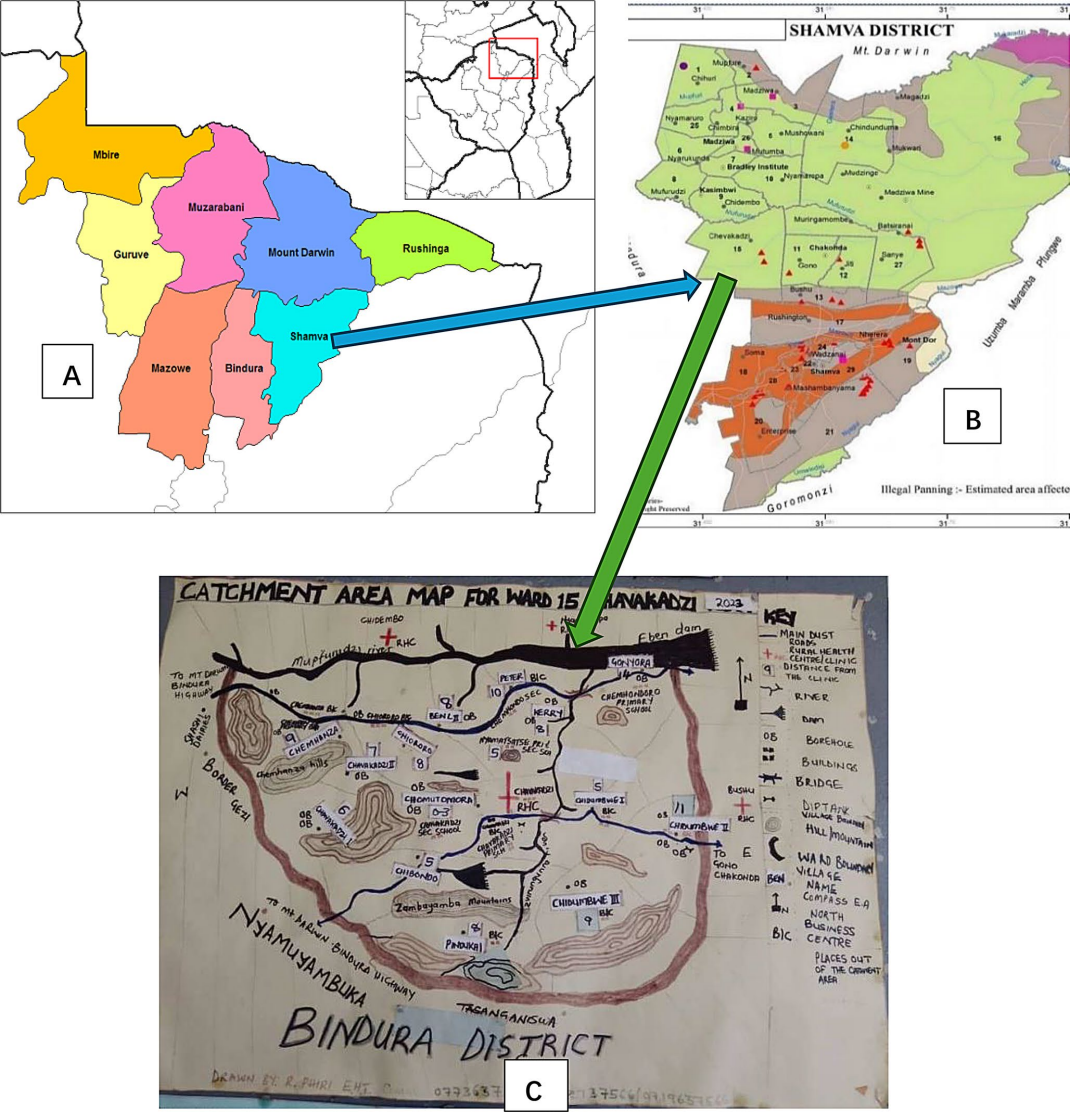
Map of Mashonaland central Province (**A**), Shamva district (**B**) and Chevakadzi ward (**C**). (Source: Ministry of Health and Child Care, Zimbabwe 2023)

Based on the above factors, Chevakadzi ward, Shamva district, Mashonaland Province in Zimbabwe (Fig. 1A, B and C) was selected as the site to carry out the pilot study. The community is in the middle west of Shamva with high rainfall and temperature in summer. The ward has a dam and several creeks and streams, which favors the transmission of SCH. The total area of Chevakadzi (ward 15) is 315km^2^. The coverage of boreholes and ventilated pit latrines is 44.0% and 51.0%, respectively. The major economic activity is farming and gardening.

### Study Design

**Fig. 2:**
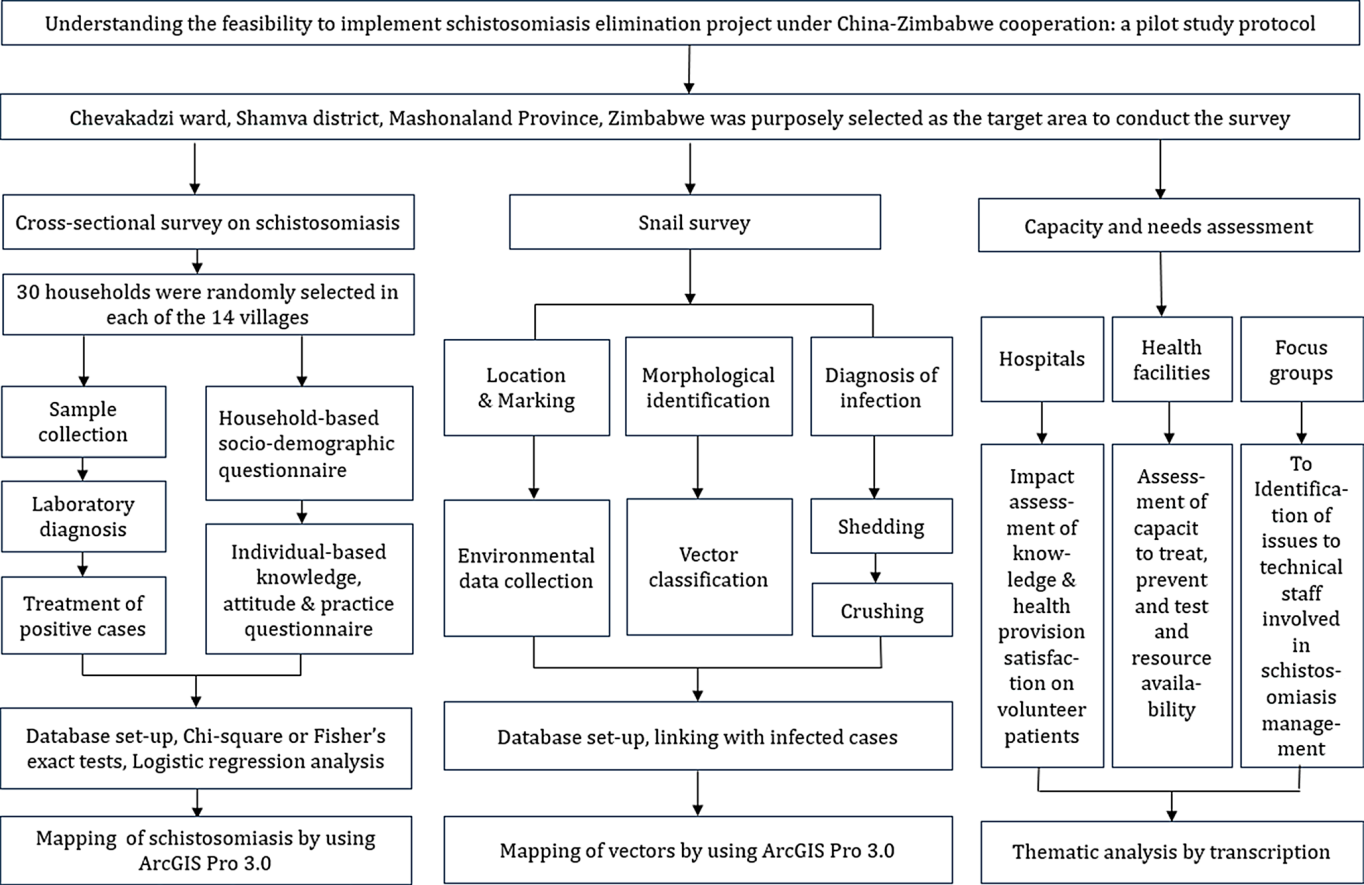
Implementation roadmap of the pilot study

The study was made up of three parts (Fig. 2). Firstly, a cross-sectional study was planned purposely in Chevakadzi to provide baseline data for SCH prevalence. Secondly, a snail survey was implemented to understand the distribution of transmission sites in the ward. Thirdly, a capacity and needs assessment was subsequently conducted to understand the environment favorable for bilateral cooperation.

### Cross-Sectional Schistosomiasis Prevalence Survey

#### Study Population

Chevakadzi has 14 villages, which includes 1,275 households and a total population of 6,470, which accounts for 4.98% of the total population in Shamva (165,641) [63]. Of those, 14.7% (952) are under 5 years old, 32.0% (2073) are 5-14 years old and 53.2% (3445) are adults (15 years old and above). The life expectancy is 49 years. The total ratio of male and female is 1:1.02.

#### Sample Size

The pilot study sample size was estimated on the basis of households. The standard error of the household sample size (n) was computed using the following formula:

SE = √[(1/mn){(1/(4k)+τ^2^ + nσ^2^}] where m standed for the number of villages in Chevakadzi; k was the number of participants that might be sampled per household; σ^2^ meant variations of prevalence between village; and τ^2^ was variations of prevalence between household within one village. Table 1 showed that the more important determinant of precision was σ; increasing n showed diminishing returns beyond 30. It was recommended to randomly sample an average of 30 households per village, which made the sample size generated by 14 villages x 30 households, equaled to 420 households. According to the Zimbabwe National Statistical Agency, an average household in Zimbabwe was 4 people, thus, the total number of participants expected to be sampled in this study was 14 villages x 30 households x 4 persons/household, which was 1,680 participants.

**Table 1:** Methodology for calculation of sample size

	*n* = 10	*n* = 30	*n* = 60	*n* = 90
σ = 0.01	0.023	0.013	0.010	0.008
σ = 0.05	0.027	0.020	0.017	0.016
σ = 0.10	0.037	0.032	0.030	0.030

#### Sampling Method

A simple random sampling method was used to select households per village in Chevakadzi. In each village, all the names of the households were written on pieces of paper and put on a plate. After thoroughly mixing, an individual randomly picked one piece of paper out of the plate until the required number of households per village was reached. This exercise was repeated in 14 villages.

#### Inclusion and Exclusion Criteria

From the selected households, members aged 1 year and above were included as participants. Critically ill and mentally challenged individuals were excluded. Household members who were not able to give specimens were also excluded.

#### Data Collection

Each participant was asked to provide approximately 50 ml of urine and 1 g of stool samples. The sample collection was performed between 1000 and 1400 h, a period when peak egg excretion is expected [64–66]. Each sample was collected, labeled in a unique container, and processed within 12 h. For young children who were not able to provide samples on their own, sample containers were provided to caregivers [22]. 

A socio-demographic questionnaire including information on family composition, socio-economic status and water contact practice was administered to the head of each household. The knowledge, attitude and practice questionnaire towards SCH were administered to individuals aged 7 years and above.

#### Diagnosis of SCH

Morbidity of *S*. *haematobium* infection was measured in two ways. For macrohaematuria, visible haematuria in the urine was detected by naked eye. For microhaematuria, two urinalysis reagent strips were used, one from Zimbabwe and another from China (URIT Medical Electronic Co., Ltd, URIT1V^8^, China). Microhaematuria was detected using a urinary dipstick reagent strip following manufacturer’s instructions. For women who indicated menstruating, blood in urine was not considered as haematuria due to infection. After that, urine samples were processed using the urine filtration technique and the prepared slides were examined under the microscope with 10X objective [64, 65]. Egg intensity was expressed as the number of *S. haematobium* eggs /10 ml of urine. Intestinal SCH (*S. mansoni)* and STH were diagnosed using the 2 stool smears prepared through the Kato Katz technique [6, 65]. A mean of *S. mansoni* eggs/STH species eggs per person was estimated by dividing a sum of eggs counted in slide A and Slide B by 2 and the result was multiplied by 24 to estimate the egg intensity of intestinal helminths (eggs/gram stool).

#### Treatment

Confirmed cases with either SCH or any of the STH were given a single dose of praziquantel and/or albendazole in tablet form following WHO recommendations [67]. The drugs used were all donated by the WHO in Zimbabwe prior to this pilot study. Participants received fruit juice and bread as an incentive.

#### Data Analysis

Data was double entered into a Microsoft Excel sheet to ensure accuracy. Then, it was cleaned and exported to Stata version 15 (Stata statistical software 2017; Stata Corp, College Station, TX). Descriptive statistics were applied to calculate the morbidity, prevalence and infection intensity of SCH and STH. Chi-square or Fisher’s exact tests were used to compare categorical variables, and logistic regression analysis was used to identify predictors of SCH infection. The mean egg counts for *S. mansoni*, and STH were compared between gender, age groups, households and villages using Fisher’s exact test. The distribution of SCH, STH, and snails were mapped using ArcGIS Pro version 3.0.

### Snail Survey

Intermediate hosts were collected to determine their distribution, infection status and diversity. Coordinates of each water contact site were recorded using a global positioning system. Environmental variables comprised of waterbody types, geographical information, contact activity and the description of site substratum were recorded. The snail sampling was carried out based on the WHO protocol [68]. A maximum of 100 scoops were performed at each water contact site and the snails collected were pooled, counted, and classified by species. Prior to testing, all snails were classified by water contact point and kept in water covered with wet cotton wool.

Snails were morphologically identified and screened for schistosome infection by cercarial shedding [69]. Emerging cercariae were inspected by both naked eye and dissecting microscope. *Bulinus globosus* and *Biomphlaria pfeifferi* are the primary intermediate snail hosts for the transmission of intestinal and urogenital SCH in Zimbabwe [69–70], hence they were deemed infected if they released bifurcated cercariae. Then the snails were checked for pre-patent infection through placing each one between clean slides and crushing it by applying pressure on the two slides. The snail shell pieces were removed and the snail was examined under the dissecting microscope at low magnification to check the presence of cercariae and/or sporocysts.

### Capacity and Needs Assessment

#### Data Collection

A patient satisfaction survey questionnaire was carried out on volunteer patients at health facilities in Shamva district to assess the impact of knowledge and health provision satisfaction. A confirmed case investigation questionnaire was administered only to positive cases to assess the efficacy of treatment. Health facility capacity survey to assess the capacity of health facilities to treat, prevent and test as well as the availability of human and equipment resources committed to SCH treatment, control and prevention was assessed at the peripheral health facility level. The focus group discussions (FGD) of SCH were conducted at national level down to the ward level. The FGDs followed a guide to explore issues around SCH. Questions were administered to homogenous technical staff involved in the management, prevention, and control of SCH. On average the sample size for each FGD was 6 participants and a total of six FGDs were conducted. Participants included multidisciplinary teams from Madziwa Rural Hospital, Chevakadzi clinic, Shamva District Hospital, Provincial Medical Directorate, Ministry of Health and Childcare Head office and WHO country office in Zimbabwe.

#### Data Analysis

Thematic analysis was used following the principles of Braun & Clarke, 2006. This required transcription of audio recordings from the FGD. This was followed by other coding phases. Transcripts were read several times to identify potential themes. The next level of analysis involved generation of initial codes. Efforts were made to retain the diversity of the initial codes while in the process of searching for higher level subthemes. Quotes that were congruent with themes were identified. Themes were outlined, clarified, named and finalized. The results were applied to identify areas for improvement in the local health system.

### Quality Control

#### Laboratory Settings, Staffing, and Training

The field laboratory was established in Madziwa Rural Hospital, Shamva District with 2 centrifuges, 1 water bath, 1 dissecting microscope, 1 fridge, and 10 microscopes.

The principal investigators were responsible for decision making, resource allocation, monitoring and reporting. The research group was composed of 4 sub-groups responsible for household surveys and sample collection and 1 team for snail survey. Each of the 4 sub-groups consisted of 5 personnel, namely: a laboratory scientist, a laboratory assistant, a nurse, a village health worker and a driver. The laboratory scientists were the team leaders for each team. Scientists and assistants from laboratory were responsible for recording household coordinates, collection of urine and stool, diagnosis of urinary and intestinal SCH, administration of the questionnaires as well as seeking informed consent at each household. The required number of laboratory technicians was calculated based on the assumption that 1 technician could examine 50 slides per day. Nurses were responsible for the blood sampling and treatment thereafter. The village health workers guided the teams into the villages and from one household to another. They also mobilized the community for sample donation. The environmental health officer and environmental health technician were responsible for guiding the snail survey team around the villages identifying water contact sites, scooping snails and recording geocoordinates. The research team members were drawn from the National Institute of Health Research, Mashonaland province, Shamva district and Chevakadzi ward.

A 6-day training workshop was organized in June, 2023. Laboratory scientists, laboratory assistants, nurses, environmental health personnel and village health workers were trained. The training program set the norms and standard for conducting field investigations and covered the following areas: objectives of the study, communication skills, interviewing techniques, filling in the questionnaire, data collection and cleaning, transport of specimens, and laboratory practices. Detailed protocols for the community-based investigation were explained to the data collectors, with a focus on the methods of obtaining informed consent, administering the questionnaire, and collecting specimens. The training was key to ensure that procedures could be properly conducted and standardized throughout the study. Lab technicians were trained with a focus on the methods of examining urine and stool samples, snail survey and needs assessment. An operational manual of the study protocol, questionnaire, informed consent, and SOPs were developed and disseminated to all implementers. Standard laboratory techniques were used to conduct the required investigations.

#### Sensitization

The Provincial Medical Director went to the Shamva district for introduction of the pilot study to the local community leadership and the community. The researchers also conducted meetings with the various groups in the target communities at various points to explain the study before implementation. The village headmen and village health workers went on to sensitize their communities. These mobilizations ended with high community turn out and buy in to the project.

On the day of survey, the purpose and details of the survey were explained to the head of each household before he or she would be requested to provide written informed consent to participate. If consent was granted, the details of the study were explained to each participant who met the inclusion criteria for informed decision to participate in the study. Household members willing to participate were requested to sign the informed consent forms or assent forms (for children aged 7-17 years). For children between the ages of 7-17 years, parental consent was initially sought followed by the child assent. For children aged 1 to 6 years, only parental informed consent was sought. Only participants with signed informed consent and/ or assent forms were enrolled into the study. Table 2 provides details of the sensitization stages from the National level to the ward level.

**Table 2:** Table mobilization and sensitization

Target	Place	Purpose/action	Representative
Ministry of Health	Harare	Briefing the pilot study and ask for support	Acting Permanent Secretary
Ministry of Primary and Secondary Education	Harare	Briefing the pilot study and ask for support	Permanent Secretary
Government of Mashonaland Central	Bindura	Introduction of the purpose of the pilot study	Health and education executives
Shamva District Hospital	Shamva	Introduction of the pilot study and its methodology	District administrators and executives, District medical officer
Communities	Chevakadzi	Introduction of the public health importance of SCH and the purpose of the visit	Ward councilor, School authorities, Village heads, Village health workers
Families	Village	Introduction of the purpose and sampling procedure	Head delegates

## Discussion

This was the first time that China implemented a SCH study in a landlocked country in Africa. The primary objective of this initiative was to test the feasibility of applying Chinese practices in SCH control and elimination. This study was expected to generate baseline information and indicators of SCH. These could inform control strategies at the sub-district level in Zimbabwe. It would also demonstrate the feasibility of carrying out such project on a larger scale. This provided a good opportunity to determine the efficiency and cost-effectiveness of integrated intervention strategies such as treatment, behavior change communication, and snail control. The study aimed to provide a platform for the Chinese counterpart to learn the different cultures and expectations of developing countries in such a cooperation.

This mix of different culture in a team brought a good start in the China-Africa initiative for the control and elimination of SCH. It also made it possible for the Chinese and Zimbabwean counterparts to learn to collaborate closely with each other and to learn from the other side’s way of doing things. For example, China’s multi-sectoral strategy that engaging agriculture, water conservancy, and public health in SCH control [46, 49, 50, 51] offered a valuable model for Zimbabwe to enhance interdepartmental coordination. In field survey methods, China’s advanced sampling and mapping techniques improved survey accuracy in Zimbabwe, while Zimbabwe’s deep understanding of local ecology helped the Chinese team better grasp context-specific transmission factors. Culturally, both countries bring unique strengths: China’s use of media for health education and Zimbabwe’s strong community engagement were complement each other to enhance public awareness and community participation. In addition, China’s model of global health cooperation emphasized close collaboration with grassroots partners, learning through implementation, and building mutual trust distinguishes it from traditional donors, which facilitated greater adaptability to local contexts.

The primary challenge encountered in this project was determining how to effectively conduct SCH survey within the existing local health system and integrate this pilot project with available local resources. This integration was crucial for ensuring the success of the survey and follow-up intervention. The insights and lessons learned from this pilot project would be instrumental in developing a locally tailored model of Chinese aid that was responsive to the unique needs of the region. Furthermore, this model has the potential to be expanded to other regions in Zimbabwe, thus contributing to broader efforts in SCH control and elimination.

Starting in 2014, China launched a tripartite initiative in collaboration with Zanzibar Island and the WHO to drive forward the control and elimination of urogenital SCH [53]. In contrast, our pilot study in Zimbabwewas an exploration of strategies for adapting both intestinal and urogenital SCH elimination efforts to a landlocked mainland African country. While our study did not explicitly focus on future scalability, its results could be a reference for initiatives across other endemic regions in Africa. The identification of future study sites might depend on multiple determinants, including the disease burden, practicality of intervention implementation, ecological variations, disparities in health systems, and pre-existing collaborative partnerships. By undertaking this pilot project, China aimed to demonstrate its commitment to global health initiatives and to share its experiences and expertise in SCH control with the international community. The successful implementation of this project could pave the way for future collaborations and joint efforts in addressing other health challenges facing Africa and beyond. The future of SCH control and elimination in Zimbabwe will depend on a carefully outlined set of evidence-based goals based on past experience, current understanding and future projections of epidemiological trends to effectively target intervention programmes to future populations.

WHO has advocated including adults and pre-school age children in MDA strategies to control SCH [71]. However, the common practice of a baseline study of SCH and STH is based on school investigation [70, 72]. This study was the first to include all-age participants from the age of 1 year old to visualize a precise estimate of SCH prevalence at the community level in Zimbabwe, which would be useful to understand the prevalence, facilitate policy formulation and update control strategies. The community-based survey would identify key findings related to epidemiological features and risk factors. Taking into consideration that SCH was characterized as a geographically focal disease, this strategy to conduct a community-level survey was in line with the WHO’s mapping strategy for NTDs in African region. The results would be used as a baseline for measuring the effects of MDA other than school-aged children as before. The information generated could be used by the program and government to advocate for policy changes and the mobilization of extra resources in support of control efforts. The results were expected to identify gaps and/or priority areas of health systems for further implemental research and exploration in a broader view. In this study, the ward of Chevakadzi was purposely selected as the study area for SCH baseline survey. Due to varied distribution of SCH in Zimbabwe [34], our findings in Chevakadzi ward would not be directly generalizable to the entire country, but they would offer insights for designing a SCH elimination program by identifying key epidemiological and implementation factors that need to be considered. The distribution and infection status of intermediate host snails play a pivotal role in local transmission. Controlling snails is one of the complementary strategies recommended by WHO for the control and elimination of SCH [68, 73]. It has been a key intervention in China’s national SCH control program. It is implemented through chemical molluscicides, forestry, agriculture, and water conservancy projects [74]. Despite the biological differences between snails in China and Africa, a recent study showed that integrating snail control with MDA helped reduce reinfection of SCH in Zanzibar [75]. China’s continuous efforts in snail control have contributed to its interruption of SCH transmission. This strongly supports the WHO’s recommendation that snail control is a key component if the goal of SCH elimination is to be achieved [76]. This study opened a window for intermediate host snail mapping which can inform vector control interventions at sub-district level in Zimbabwe.

There were some limitations of the study design. Firstly, inadequate research funding constrained the scope of the study to a single community, necessitating a delicate balance between resource availability and the potential research impact. Secondly, due to intricate regulatory and ethical hurdles for cross-border drug registration, Zimbabwean SCH patients were unable to promptly access Chinese-manufactured praziquantel. Thirdly, the study was conducted during June and July, which is the cold, dry season in Zimbabwe. This could have influenced shrinking of waterbodies especially in seasonal water sources, and reduction in snail reproduction. In addition, WHO targets the elimination of SCH as a public health problem by 2030, thus diagnostic tests in low transmission areas are in need. In this regard, more sensitive diagnosis such as point-of-care circulating cathodic antigen test should be considered, which is as recommended by WHO. However, due to some technical reasons [77–79], as well as the availability of the diagnostic tool, it was not included in this pilot study. These challenges underscored the importance of comprehensive planning and strategic resource allocation in global health cooperation initiatives, particularly in the context of infectious disease control where timely and effective intervention was crucial.

In summary, this study not only explored the transfer of China’s experience in controlling and eliminating SCH to African countries but also provided a novel perspective for China-Africa public health collaboration. It held significant reference value for advancing African countries’ progress towards the WHO’s Universal Health Coverage (UHC) initiative and the elimination of NTDs.

## Electronic Supplementary Material

Below is the link to the electronic supplementary material.

Supplementary Material 1

## Data Availability

No datasets were generated or analysed during the current study.

## Abbreviations

SCH: Schistosomiasis
NTDs: Neglected Tropical Diseases
PC: Preventive Chemotherapy
WHO: World Health Organization
MDA: Mass Drug Administration
SAC: School-aged Children
FOCAC: Forum on China-Africa Cooperation
STH: Soil Transmitted Helminthiasis
FGD: Focus Group Discussion
