# Supplementary figures and images for: Understanding the Feasibility to Implement Schistosomiasis Elimination Project Under China-Zimbabwe Cooperation: A Pilot Study Protocol
Source: J Epidemiol Glob Health. 2025 May 26;15(1):75. doi: 10.1007/s44197-025-00418-6 (PMC12106187; doi:10.1007/s44197-025-00418-6)

### Household Survey

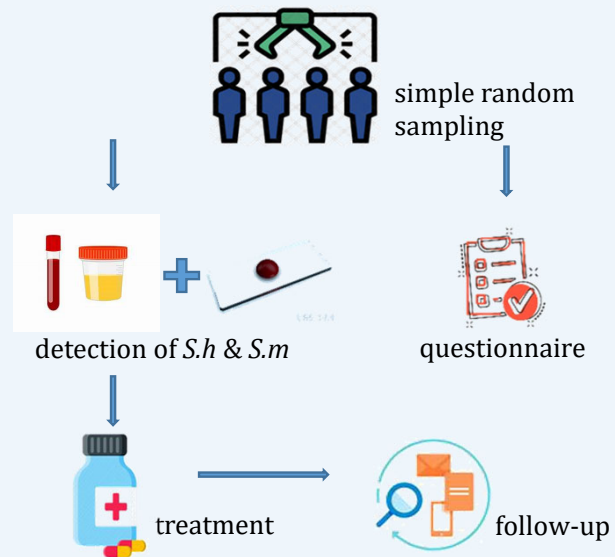

### Snail Survey

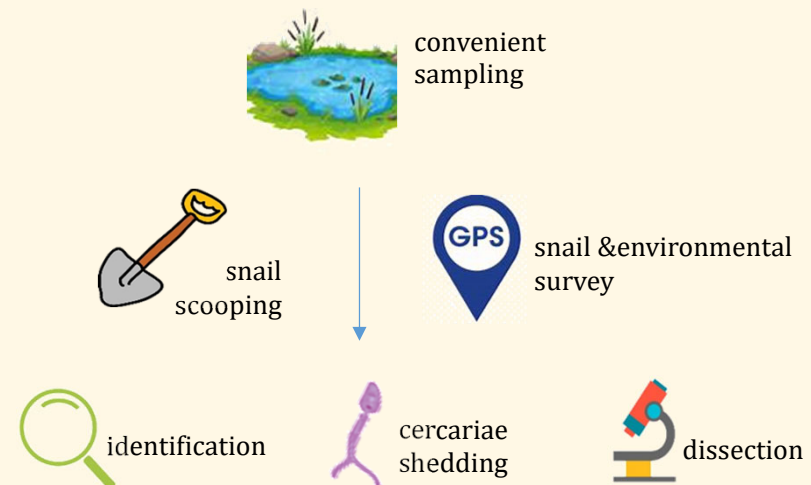

### Capacity & Needs Assessment

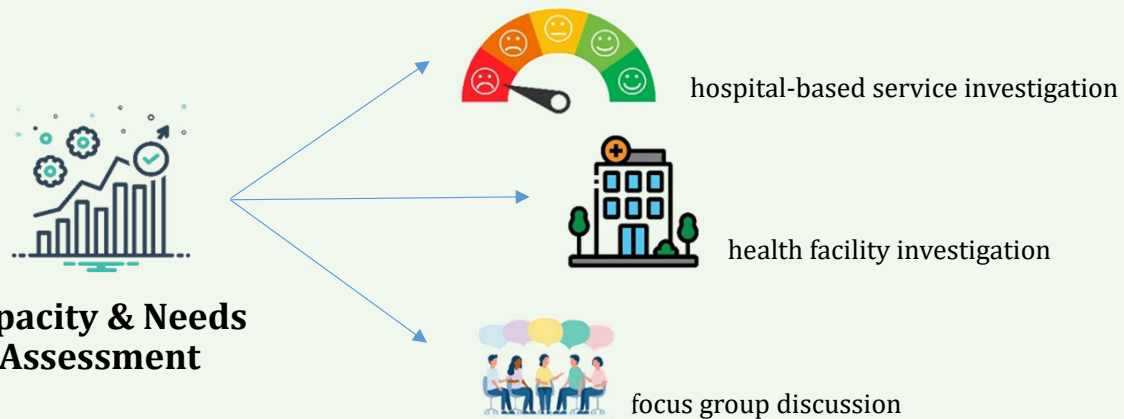

Supplement: Supplementary file 1 — Supplementary Material 1 [file 44197_2025_418_MOESM1_ESM.pdf]
